# Osteogenic differentiation of mesenchymal stem cells is enhanced in a 45S5-supplemented β-TCP composite scaffold: an *in-vitro* comparison of Vitoss and Vitoss BA

**DOI:** 10.1371/journal.pone.0212799

**Published:** 2019-02-27

**Authors:** Fabian Westhauser, Maria Karadjian, Christopher Essers, Anne-Sophie Senger, Sébastien Hagmann, Gerhard Schmidmaier, Arash Moghaddam

**Affiliations:** 1 Center of Orthopedics, Traumatology, and Spinal Cord Injury, Heidelberg University Hospital, Heidelberg, Germany; 2 ATORG—Aschaffenburg Trauma and Orthopedic Research Group, Center for Trauma Surgery, Orthopedics, and Sports Medicine, Klinikum Aschaffenburg-Alzenau, Aschaffenburg, Germany; Northeastern University, UNITED STATES

## Abstract

Since the amount of autologous bone for the treatment of bone defects is limited and harvesting might cause complications, synthetic bone substitutes such as the popular β-tricalcium phosphate (β-TCP) based Vitoss have been developed as an alternative grafting material. β-TCPs exhibit osteoconductive properties, however material-initiated stimulation of osteogenic differentiation is limited. These limitations might be overcome by addition of 45S5 bioactive glass (BG) particles. This study aims to analyze the influence of BG particles in Vitoss BA (20 wt% BG particles with a size of 90–150 μm) on osteogenic properties, cell vitality and cell proliferation in direct comparison to Vitoss by evaluation of the underlying cellular mechanisms. For that purpose, Vitoss and Vitoss BA scaffolds were seeded with human mesenchymal stem cells (MSC) and underwent osteogenic differentiation *in-vitro* for up to 42 days. Cell vitality, proliferation, and osteogenic differentiation were monitored by quantitative gene expression analysis, determination of alkaline phosphatase activity, PrestoBlue cell viability assay, dsDNA quantification, and a fluorescence-microscopy-based live/dead-assay. It was demonstrated that BG particles decrease cell proliferation but do not have a negative impact on cell vitality. Especially the early stages of osteogenic differentiation were significantly improved in the presence of BG particles, resulting in earlier maturation of the MSC towards osteoblasts. Since most of the stimulatory effects induced by BG particles took place initially, particles exhibiting another surface-area-to-volume ratio should be considered in order to provide long-lasting stimulation.

## Introduction

Bone defect treatment belongs not only to the most challenging fields in orthopedic surgery, but is also one of the most relevant clinical procedures in modern medicine and will be of increasing importance due to the demographic development [1, 2]. Since the current clinical gold standard—iliac crest bone grafting—can only provide a certain amount of bone grafting material and is potentially followed by donor site complications, the search for appropriate biomaterials that can either reduce or replace the use of autologous tissues is in the spotlight of current orthopedic research [3, 4]. The development of synthetic bone grafts is an especially attractive and important field, since synthetic materials can not only be produced in large quantities but can also be tailored to meet specific needs in their anticipated field of application [2, 4, 5].

Currently, the most frequently used synthetic bone grafting materials are calcium phosphates (CaPs) such as tricalcium phosphates (TCP; Ca_3_(PO_4_)_2_) [4, 6, 7]. TCP appears in different polymorphs and is mostly used as β-TCP in orthopedic applications [4, 8]. Porous β-TCPs are osteoconductive, safe in clinical use and closely mimic the anorganic portion of bone [7, 9]. However, β-TCPs show certain limitations such as poor bonding properties and restricting cell attachment and connection to surrounding tissues caused by a comparably low surface reactivity resulting in negative effects on osteogenic properties [10, 11]. Furthermore, when used alone, β-TCP induces limited osteogenic differentiation of mesenchymal stem cells (MSC), which are osteoblast precursors and of certain relevance in bone defect consolidation [12, 13].

A promising alternative synthetic bone grafting material is the 45S5 (45% SiO_2_, 24.5% Na_2_O, 24.5% CaO, 6% P_2_O_5_, in wt%) bioactive glass (BG) that was developed by Hench and coworkers in the late 1960s [14–16]. The 45S5 BG releases its ionic constituents upon implantation *in-vivo* or in contact with (body) fluids–this process is followed by hydroxycarbonate apatite formation on the surface of the BG structures, not only allowing for strong bonding to tissues but also providing favorable conditions for stem cell and osteoblast attachment, supporting bone formation on the BG-cell interface [17–19]. Along with the strong attachment of cells to the BG surface, which stimulates bone formation, the controlled ion release from the BG structure promotes the osteogenic differentiation of stem cells. The development and activity of osteoblasts is also influenced in a positive way, making 45S5 BG a Class-A biomaterial [14, 17, 19–21]. However, a possible limitation for the use of 45S5 BG in tissue engineering and bone defect reconstruction is the dramatic increase of pH caused by the release of Sodium-ions from the glass structure which can be harmful for cells and tissues [22, 23]. One of the key features required to provide bone formation within bone substitutes, regardless of the scaffold material, is a three-dimensional porous inner structure [24, 25]. Several approaches have been developed to produce porous scaffolds based on 45S5 BG [20, 25–27]. However, the crystallization of the BG structure that takes place during heat-treatment prior to 3D-modelling decreases mechanical strength, limiting not only osteogenic properties but also the application in load-bearing parts of the skeleton [28].

By designing composite materials consisting of β-TCPs and BGs, the advantages of both materials might be combined and/or limitations might be reduced. For example, an improvement of surface reactivity of β-TCP scaffolds can be achieved by addition of BG: the BG can provide superior attachment of cells followed by increased stimulation of osteogenic differentiation [6]. Furthermore, it was shown that the addition of BGs as a second phase improved the mechanical properties of β-TCP based scaffolds [6, 29].

So far, there is a limited amount of biological data analyzing the very basic osteogenic properties of β-TCP BG composite scaffold materials [5, 30, 31]. Furthermore, there is poor experimental evidence concerning the influence of 45S5 BG particles added to β-TCP based scaffolds from studies that directly compare β-TCP scaffolds to BG-supplemented β-TCP based scaffolds regarding osteogenic properties [6, 30, 31]. However, some of these composite scaffold materials are already in clinical use: one of the most frequently used β-TCP based bone graft materials with more than 600,000 implantations worldwide is the β-TCP based Vitoss (Stryker, Kalamazoo, USA) that can also be purchased as Vitoss BA [9, 32]. Vitoss BA Foam Pack is a composite scaffold material containing β-TCP particles bonded on a collagen matrix (Vitoss) supplemented with 20 wt% 45S5 BG particles with a particle size of 90–150 μm [6, 9, 32]. It has previously been demonstrated that consolidation of a metaphyseal bone defect in dogs is enhanced when treated with Vitoss BA compared to defects treated with Vitoss [33]. Furthermore, mechanical stability of the bone returned faster to native strength in the defects treated with Vitoss BA [33]. However, the influence of the 45S5 BG particles in Vitoss BA on cell vitality, proliferation and osteogenic differentiation are not yet investigated and warrant for clarification in order to provide a better understanding of the material’s properties and the underlying cellular mechanisms.

Therefore, in this study, the influence of BG particles in Vitoss BA was compared to Vitoss bone graft material in order to assess osteogenic differentiation and impact on cell vitality and proliferation. Both scaffold types underwent seeding with human bone marrow-derived MSC and were then cultivated in osteogenic differentiation medium (ODM) for up to 42 days *in-vitro*. Analysis of initial morphology was conducted by micro-computed tomography (mCT); osteogenic performance and cell vitality were monitored by quantitative real-time gene expression analysis (qPCR), determination of alkaline phosphatase activity (ALP), PrestoBlue cell viability assay and dsDNA quantification assay, and by fluorescence-microcopy-based live/dead-assay. This study was executed in order to provide directly related basic experimental evidence to improve understanding and to evaluate the influence of 45S5 BG particles in Vitoss BA on cell vitality and the osteogenic properties of Vitoss.

## Materials and methods

### Study ethics and patient demography

MSC were harvested of n = 10 donors that underwent treatment for hypertrophic non-union at Heidelberg University Hospital. All patients gave written consent prior to bone marrow harvesting that took place during non-union surgery. The average age of the patients was 45.5 years (range: 31–61, median: 45). 2 female and 8 male patients were included. We did not match to risk factors that might influence MSC-quality in order to provide realistic conditions by using cells of a heterogeneous collective which improves transferability of the clinical routine situation into this experimental *in-vitro* setting and vice versa. The study was approved by the ethics committee of the Medical Faculty of the University of Heidelberg (S-443/2015).

### MSC characterization, isolation, and cultivation

Isolation, cultivation, expansion and characterization of MSC from bone marrow were conducted as published previously, following standardized protocols [34, 35]. Stem cell criteria were defined according to the consensus of the international society for cellular therapy by plastic adherence, trilineage differentiation, and by analysis of specific surface characteristics using fluorescence-labeled cell sorting (FACS) as published previously [34, 36].

### Scaffold fabrication, cell seeding, and osteogenic differentiation protocol

**Fig 1:**
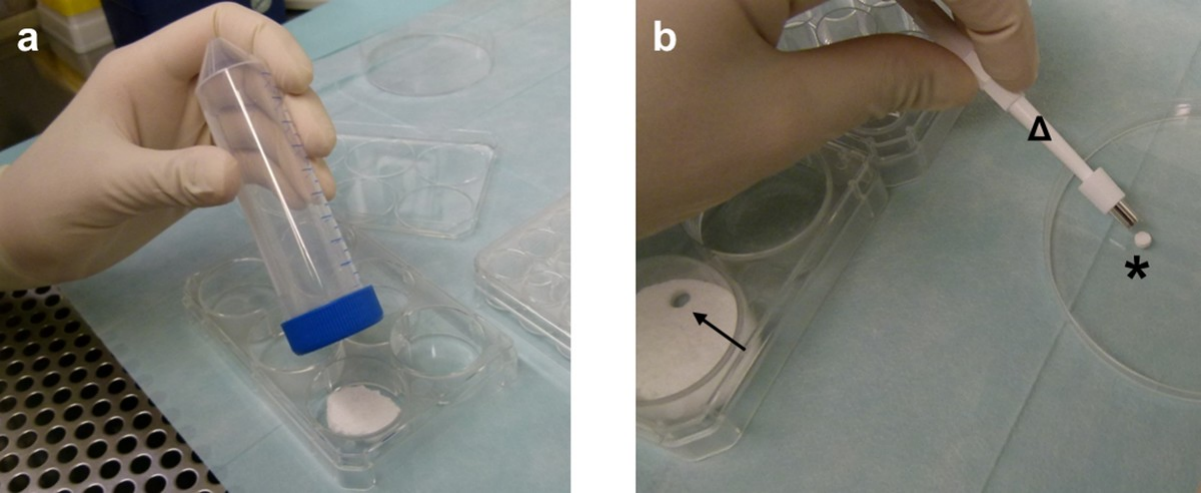
Scaffold production. After the wetted foam pack was kneaded according to the manufacturer’s recommendations, it was pressed into one well of a 6-well plate using a lid of a 50 ml tube to achieve equal distribution of the foam in the well (a). A 5 mm biopsy punch (b, Δ) was used to obtain cylindrically shaped scaffolds (b, *) from the compressed foam (b,→).

Both Vitoss and Vitoss BA (Stryker, Kalamazoo, USA) were provided as Foam Packs with a volume of 2.5 cm^3^. The Foam Packs were treated according to the manufacturer's instructions. In order to obtain standardized scaffolds, wetted Vitoss or Vitoss BA foam was placed into a well of a 6-well plate (Thermo Fisher Scientific, Dreieich, Germany) with a defined surface area of 9.6 cm^2^. The foam was then pressed with the lid of a sterile 50 ml tube (Greiner Bio One, Frickenhausen, Germany) until the whole plate was equally covered with Vitoss or Vitoss BA at a height of approximately 2.7 mm (Fig 1A). By using a biopsy punch (Stiefel, Wächtersbach, Germany) with an internal diameter of 5 mm, standardized cylindrically shaped scaffolds were obtained (Fig 1B). The procedure was performed under sterile conditions. The scaffolds were dried for at least 24h in a sterile environment and were then seeded with MSC following established drop-seeding protocols [25]. In short, 250,000 MSC were diluted in 10 μl PBS (Thermo Fisher Scientific, Dreieich, Germany) and placed on the top of the respective scaffolds located in 24-well plates (Thermo Fisher Scientific, Dreieich, Germany). The scaffolds underwent incubation at 37°C for 30 min to allow the cells to adhere before 1 ml osteogenic differentiation medium (ODM) was added.

The ODM was composed of 25 mM DMEM high-glucose with L-Glutamine (Thermo Fisher Scientific, Dreieich, Germany), 10% fetal calf serum (Thermo Fisher Scientific, Dreieich, Germany), Penicillin/Streptomycin 100 mg/l (Merck, Darmstadt, Germany), Amphotericin B 2.5 mg/l (Merck, Darmstadt, Germany), dexamethasone 0.1 μM (Sigma Aldrich, Steinheim, Germany), ascorbic acid-2-phosphate 2.5 mg/l (Sigma Aldrich, Steinheim, Germany), beta glycerophosphate 10 mM (Merck, Darmstadt, Germany).

The scaffolds were incubated for up to 42 days at 37°C, 5% CO_2_. Medium was changed 24 h after the start of the incubation period and then twice per week.

### General experimental design

The MSC-seeded Vitoss and Vitoss BA scaffolds were subjected to the same experimental protocol for a total incubation time of 42 days. To provide comprehensive understanding of the scaffolds’ influences on cell vitality, cell proliferation, and osteogenic performance, several methods were used at defined time points. An overview about the general experimental design and the used assays is provided in Table 1.

**Table 1:** Overview regarding the used assays and observation time points.

Assay	D0	D1	D7	D14	D21	D42
mCT	X					
ALP activity		X	X	X	X	X
PrestoBlue		X	X	X	X	X
dsDNA		X	X	X	X	X
Live/dead-assay					X	X
qPCR		N	X	X	X	X
pH		X	X	X	X	X

D: day of incubation, X: assay conducted. mCT-evaluation was performed before the incubation period started (D0). In order to obtain a baseline level and to provide normalization (N) for the subsequent assays, gene expression (qPCR)-analysis was performed after 24 hours of incubation (D1).

The MSC metabolism after 24h of incubation was considered as a baseline for the following osteogenic differentiation and was therefore used as normalization for qPCR assays [37]. Each assay was performed in quadruplicate. This means that four identical scaffolds were provided per assay, donor, observation time point, and scaffold type.

### Evaluation techniques

#### Micro-computed tomography (mCT)

mCT analysis was performed to obtain basic data concerning the total volume of the scaffolds (SV) in order to check if the scaffold production process allowed for standardized fabrication of scaffolds. Furthermore, for visualization purposes, both two-dimensional (2D) sections and three-dimensional (3D) models of scaffolds of both types were shown.

mCT scans were conducted with a SkyScan 1076 (Bruker micro-CT, Kontich, Belgium) using the following specifications: tube current 120 μA, integration time 700 ms, voltage 90 kV, pixel size 9 μm, 0.5 mm Aluminum filter. The RAW datasets were reconstructed with NRecon (Version 1.6.9.8, Bruker micro-CT, Kontich, Belgium) as published previously [20, 25, 38]. The reconstructed datasets were analyzed by Heidelberg-mCT-Analyzer following published protocols [20, 25, 38, 39]. For 3D visualization, CTVox (Version 3.1, Bruker micro-CT, Kontich, Belgium) was used: one representative RAW dataset each of both scaffold types was opened and grey-value-aligned and converted into 3D models using the onboard algorithms of the program.

#### ALP activity assay

Alkaline Phosphatase (ALP) is one of the most reliable markers for osteogenic differentiation, since it is produced by osteogenic cells such as osteoblasts [34, 35]. The scaffolds were washed with 1x PBS and then kept at -80°C in dry conditions until further use. The frozen MSC-seeded scaffolds underwent homogenization using a Polytron PT 1200 E benchtop homogenizer (Kinematica, Lucerne, Switzerland) in 1% Triton buffer (Sigma Aldrich, Steinheim, Germany). The homogenate was centrifuged and the supernatant was added to a solution of para-Nitrophenylphosphate (p-NPP) (Sigma Aldrich, Steinheim, Germany) for 90 min. The ALP converts p-NPP to para-Nitrophenol (p-NP) causing a change of color to yellow. The extinction of p-NP which corresponds to the ALP activity was measured photometrically at 405 nm with a reference wavelength of 490 nm using a MRX Microplate Reader (Dynatech Laboratories, Stuttgart, Germany). The ALP activity was normalized to the dsDNA content of each sample, since the amount of dsDNA is directly correlated with the number of cells.

#### Cell proliferation assay (dsDNA-assay)

Since the amount of dsDNA directly correlates with the number of the mononuclear MSC, cell proliferation activity can be analyzed via measuring dsDNA content. The amount of dsDNA of each sample was determined using the Quant-iT PicoGreen dsDNA Assay Kit (Thermo Fisher Scientific, Dreieich, Germany) according to manufacturer’s instructions. The dsDNA was measured from the supernatant of the centrifuged scaffold homogenates that were prepared for ALP-activity measurement. The samples containing 1% Triton were diluted in a ratio of 1:10 using 1x TE-Buffer provided with the kit in order to reduce the Triton concentration to the recommended maximum of 0.1%. A total volume of 100 μl of the diluted sample was used for dsDNA measurement. The dsDNA-content was measured using a Wallac 1420 Victor2 Microplate Reader (Perkin Elmer, Rodgau Jügesheim, Germany). Samples were excited at 485 nm and fluorescence emission intensity was measured at 535 nm. The data was used to determine cell proliferation over the time of incubation and for normalization of the ALP activity.

#### Cell viability assay (PrestoBlue assay)

Cell viability was assessed using the PrestoBlue assay (Thermo Fisher Scientific, Dreieich, Germany). PrestoBlue contains the blue dye resazurin which is reduced to the red dye resorufin in the presence of living cells. The percentage reduction of the PrestoBlue dye positively correlates with cell viability. The samples were prepared according to manufacturer's instructions: 100 μl PrestoBlue dye was added to 900 μl of ODM containing one scaffold each. After 90 min of incubation at 37°C, the color change was determined by measuring the absorbance using a UV-1600 PC spectrophotometer (VWR International, Darmstadt, Germany) at 570 nm with a reference wavelength of 600 nm. The reduction was normalized to a blank sample containing an unseeded scaffold (one per group), ODM, and PrestoBlue dye. After the measurement, the scaffolds were washed with 1x PBS and then used for further incubation.

#### Live/dead-staining via fluorescence microscopy

To determine cell survival on the scaffolds, a live/dead-staining with fluorescein diacetate (FDA) and propidium iodide (PI) was performed at D21 and D42. FDA is able to pass cell membranes and is converted to the green fluorescent compound fluorescein [40]. The red fluorescent PI cannot pass cell membranes and intercalates the DNA in the nucleus of membrane-compromised cells, which is a sign of cell death. After rinsing the scaffolds with 1x PBS, they were incubated with 2 μg/ml FDA (Sigma Aldrich, Steinheim, Germany) for 15 min at 37°C and then again rinsed with 1x PBS before incubation with 20 μg/ml PI (Thermo Fisher Scientific, Dreieich, Germany) solution for 2 min at room temperature. The scaffolds were rinsed with 1x PBS again and then kept in PBS for immediate evaluation under an Olympus IX-81 inverted fluorescence microscope (Olympus, Hamburg, Germany).

#### RNA isolation and quantitative real-time PCR (qPCR)-analysis

Gene expression analysis was performed in order to determine the level of osteogenic differentiation of the MSC. Total RNA was isolated from the scaffolds using a TRIzol (Thermo Fisher Scientific, Dreieich, Germany) based protocol following the manufacturer’s instructions with some modifications: the scaffolds were washed twice with 1x PBS before homogenization in 1ml TRIzol using the benchtop homogenizer and then incubated at 4°C for 18 h. After incubation, RNA-isolation was undertaken using the PureLink RNA Mini Kit (Thermo Fisher Scientific, Dreieich, Germany) following the manufacturer's instructions. A total amount of 100 ng RNA per sample was transferred to cDNA synthesis using the SensiFast cDNA Synthesis Kit (Bioline, Luckenwalde, Germany). For real time qPCR the SensiFast SYBR LoRox Kit (Bioline, Luckenwalde, Germany) according to manufacturer’s instructions for selected osteogenic genes (primers shown in Table 2) was used. Gene expression was evaluated with the delta delta Ct method using the gene expression of D1 as a calibrator and normalized to internal YWHAZ expression.

**Table 2:** Primers used for qPCR analyses.

	Forward (5’ → 3’)	Reverse (5’ → 3’)
YWHAZ	TGCTTGCATCCCACAGACTA	AGGCACAATGACAGACCA
RUNX2	TAGGCGCATTTCAGGTGCTT	TGGCAGGTAGGTGTGGTAGT
SPP1	GCTAAACCCTGACCCATCTC	ATAACTGTCCTTCCCACGGC
COL1A	GCAAGAACCCCAAGGACAAGAG	TCGTGCAGCCATCGACAGTGAC

14-3-3 protein zeta/delta (YWHAZ), Runt-related transcription factor 2 (RUNX2), secreted phosphoprotein 1 (SPP1), collagen type I alpha 1 chain (COL1A).

#### pH-measurement

In order to monitor pH-alterations induced by BG particles as a correlate of ion release and bioactivity, the pH was measured from the ODM containing either Vitoss or Vitoss BA scaffolds. 1 ml ODM was transferred to 15 ml tubes (Greiner Bio One, Frickenhausen, Germany) and underwent immediate pH measurement using a benchtop pH-meter (PB-11-P10.1M, Sartorius, Göttingen, Germany).

### Statistics

Statistical analyses were conducted with IBM SPSS Statistics (Version 25; IBM, Mannheim, Germany) and graphs were created using GraphPad Prism (Version 7, GraphPad Software, La Jolla, USA). Before further analysis, values were tested for normal distribution by Shapiro-Wilk-test. According to the result of the test, normally distributed samples were tested with the paired T-Test; not normally distributed samples were tested using the Wilcoxon signed-rank test. Results were described as statistically significant for p<0.05. Unless otherwise stated, differences mentioned in the text are non-significant. Values are shown as rounded means with the standard error of the mean where applicable.

## Results

### Cell characterization

**Fig 2:**
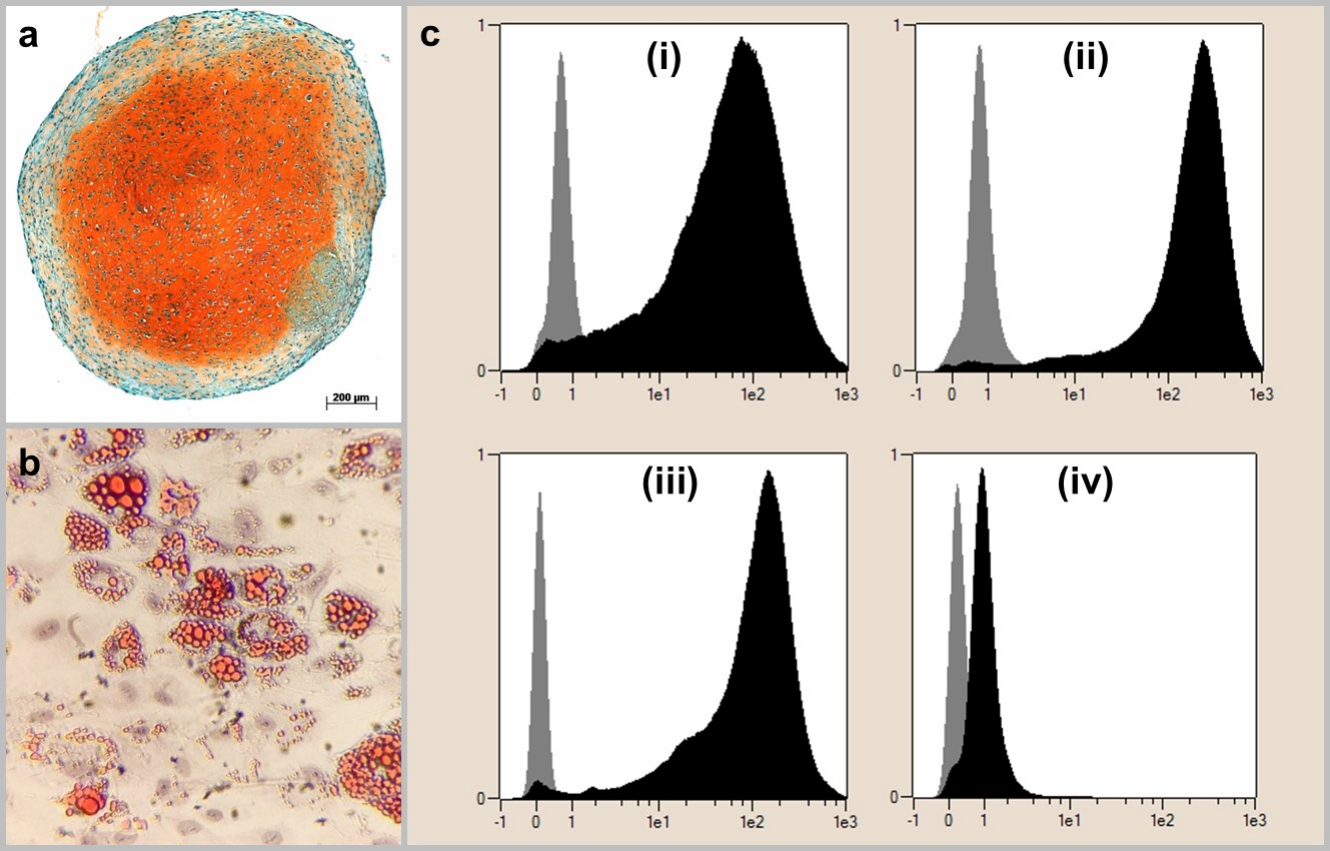
Definition of the cell population as MSC. The cell populations were able to differentiate into chondrogenic lineage as demonstrated by the orange-colored extracellular chondrogenic matrix in Safranin O/Fast Green staining (a) and in adipogenic lineage, as shown by red-colored vacuoles in Oilred-O staining (b). Furthermore, the cells exhibit the MSC-specific surface characteristics (c): (i) corresponds to CD90, (ii) to CD105, (iii) to CD73 and (iv) to the negative control composed of CD14, CD20, CD34 and CD45. The gray bar in the left part of each graph shows the results of the isotype control being labeled with murine antibodies whilst the black bar on the right shows the results of human MSC being labeled with human antibodies. The x-axis shows the fluorescence intensity in logarithmic scale, the y-axis represents a height-normalized linear scale of cell number. Results are shown representatively for one donor.

The isolated cells exhibited the required features to be defined as MSC: all cells showed plastic adherence and the ability for chondrogenic and adipogenic differentiation (Fig 2A and 2B). The ability for osteogenic differentiation was one of the main interests of this study and results are therefore shown in the following paragraphs in detail. In FACS-analysis, >95% of cells detected positive for CD90, CD73 and CD105 and <2% of cells detected positive for CD14, CD20, CD34 and CD45 (Fig 2C).

### Scaffold morphology

The total scaffold volume was 31.86 mm^3^ (±0.39 mm^3^) for Vitoss and 34.58 mm^3^ (±0.49 mm^3^) for Vitoss BA scaffolds; differences were not significant representing standardization of the production process.

**Fig 3:**
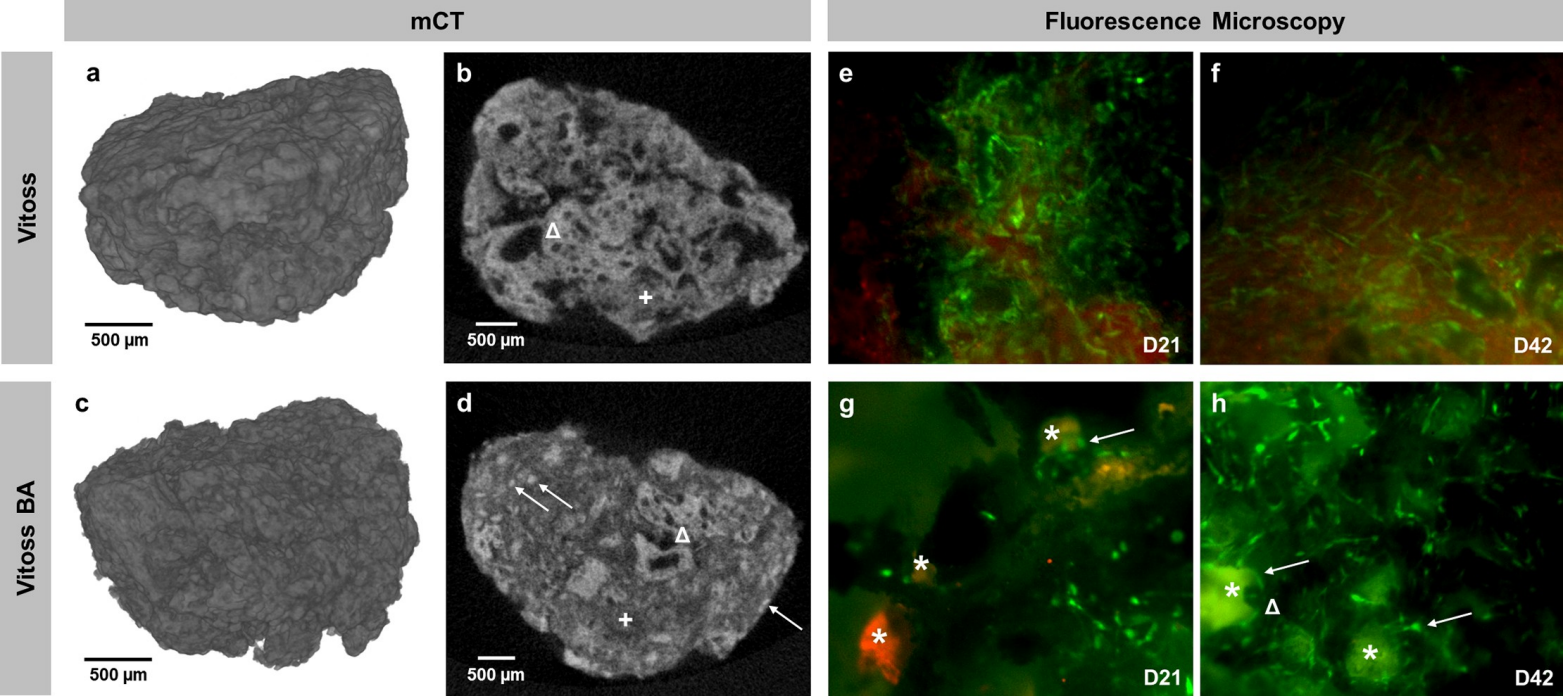
**3D-mCT reconstruction and 2D-mCT slice of one representative Vitoss (a, b) and Vitoss BA scaffold (c, d). Representative fluorescence staining of a Vitoss scaffold after 21 (e) and 42 days (f) of incubation, respectively a Vitoss BA scaffold after 21 (g) and 42 days (h).** The distribution of BG particles within the 2D scaffold structure (d, →) can be anticipated by scrolling through a stack of 2D-images: poreless, high-density particles with a size of 90–150 μm can be identified as BG. Both scaffold types consist of porous β-TCP granules (b and d, Δ) and a collagen matrix (b and d, +). Scale bars refer to 500 μm. In fluorescence staining, the green-stained intact cells dominate in both groups. Several BG particles remained intact at D21 (g, *) and some cells grew with contact to the particles (g and h, →), partially forming clusters (h, Δ). On D42 the fluorescence intensity of the BG particles decreased (h, *) compared to intensity at D21. Magnification: 40-fold.

Fig 3 shows 3D models of both scaffold types as well as representative 2D sections (Fig 3A–3D). The collagen matrix bonds the β-TCP-parts together forming a porous scaffold. In Vitoss BA-scaffolds, the collagen matrix also contains the BG particles (Fig 3D).

### Cell viability and pH alterations

#### Fluorescence microscopy

Qualitative assessment of viable and dead cells was performed in order to assess the biocompatibility of the scaffolds. Surfaces of scaffolds of both groups were dominated by viable cells after 21 (Fig 3E–3G) and 42 days of incubation (Fig 3F–3H). BG particles were detectable after 21 days of incubation (Fig 3G); whilst the fluorescence intensity of the particles decreased after 42 days in culture (Fig 3H). In Vitoss BA scaffolds, some cells grew in contact with the BG particles (Fig 3G), after 42 days some of the particles were surrounded by cell clusters (Fig 3H).

#### Cell viability and proliferation

**Fig 4:**
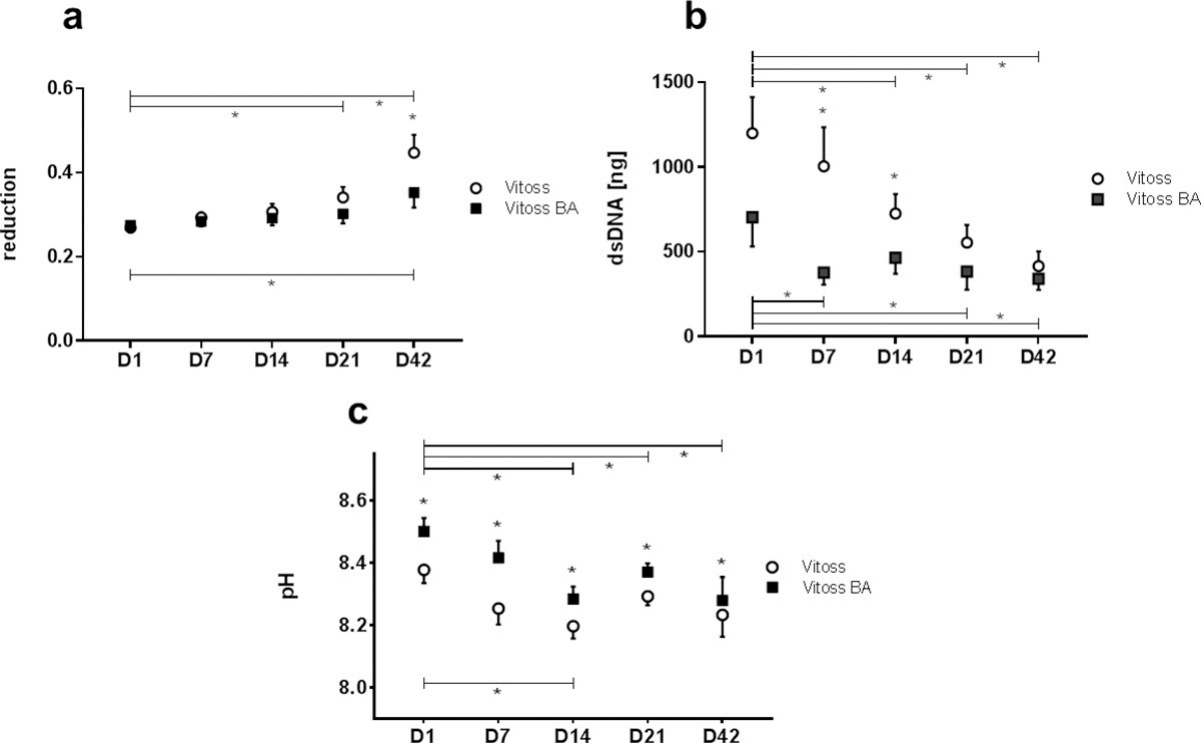
**PrestoBlue reduction as correlate of cell vitality (a), cell proliferation measured via dsDNA content (b) and pH alteration (c) during the incubation time (D1-D42)**. Values are shown as means with standard error of the mean. (*) indicates significant differences.

Percentage reduction of PrestoBlue dye, correlating with cell viability, of Vitoss and Vitoss BA scaffolds did not show significant differences and sustained a stable level during the first three weeks of cell culture (Fig 4A). On D42, Vitoss scaffolds showed significantly higher reduction (p = 0.043), indicating greater cell viability than Vitoss BA. Both Vitoss and Vitoss BA scaffolds’ percentage reduction increased over time, presenting significantly higher values on D42 compared to D1 (Vitoss: p = 0.003; Vitoss BA: p = 0.040).

The dsDNA quantification assay was performed in order to assess cell proliferation. The dsDNA amount in both scaffold types decreased significantly over time from 1199 ng in Vitoss and 703 ng in Vitoss BA scaffolds (D1) to 415 ng in Vitoss (p = 0.005) and 341 ng in Vitoss BA (D42; p = 0.013; Fig 4B). The decrease was with 65% stronger in Vitoss scaffolds compared to the 52% decrease in Vitoss BA-scaffolds. Furthermore, the dsDNA amount remained more or less stable in Vitoss BA scaffolds in the period from D7 to D42, whilst the decrease continued steadily in the Vitoss group.

#### pH alterations

pH values of Vitoss BA scaffolds were significantly higher than those of Vitoss scaffolds at all measured time points (Fig 4C). The pH of the medium containing Vitoss scaffolds was more or less stable during the incubation period whereas the pH of ODM containing Vitoss BA scaffolds decreased significantly (p = 0.026) from 8.50 on D1 to 8.28 on D42. The maximum difference of 0.16 between both groups was detectable on D7. pH values of both groups approach each other during incubation time. Whilst the difference on D1 was 0.12, the remaining pH-difference on D42 was 0.05.

### Osteogenic differentiation

#### ALP activity

**Fig 5:**
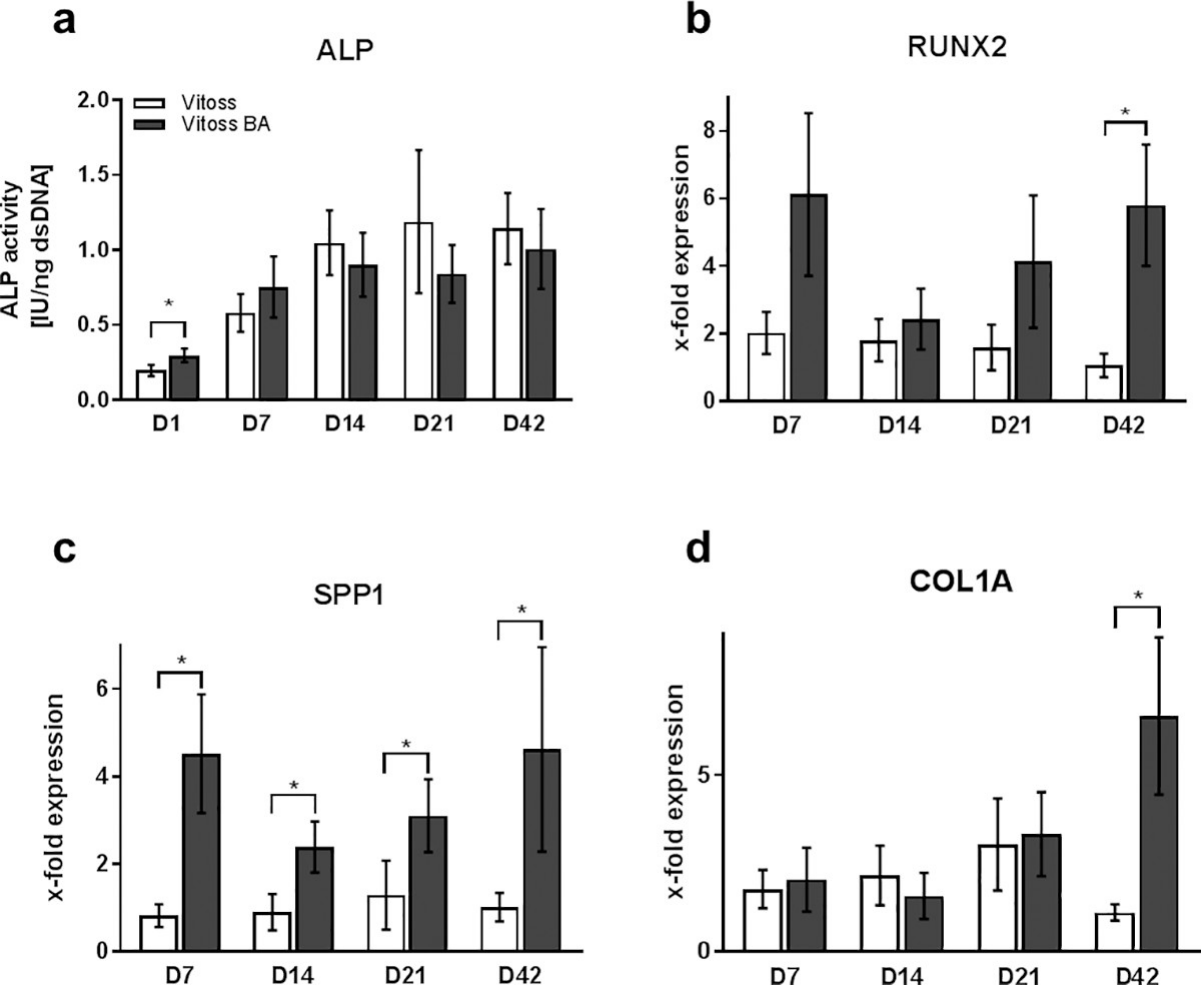
Correlates of osteogenic differentiation. ALP activity (a) and quantitative gene expression of RUNX2 (b), SPP1 (c), and COL1A (d) during the incubation time (D1-D42). Since ALP activity was significantly higher at any observation point for D7, D14, D21, and D42 when compared to D1 in both groups, significances are not indicated in the figure for reasons of clarity. Gene expression was normalized to gene expression on D1 and is therefore shown as x-fold expression. Values are shown as means with standard error of the mean. (*) indicates significant differences.

ALP activity increased significantly from D7 to D42 compared to D1 for scaffolds of both groups correlating with progressing osteogenic differentiation (Fig 5A). ALP activity in Vitoss scaffolds increased significantly from D1 to D7 (p = 0.005) and from D7 to D14 (p = 0.005) and then remained stable until D42. ALP activity in Vitoss BA increased by 300% during the first two weeks of cell culture and also maintained a steady state beginning from D14. Until D7, ALP activity of Vitoss BA scaffolds was higher, with a significant relative difference of 30% at D1 (p = 0.007) compared to ALP activity in Vitoss scaffolds. By D7, there were no significant differences in ALP activity between Vitoss and Vitoss BA scaffolds.

#### qPCR

RUNX2 expression of cell-seeded Vitoss scaffolds remained consistent at early time points and then decreased significantly (p = 0.017) from D7 to D42 by 48% (Fig 5B). RUNX2 expression in Vitoss BA peaked at D7 (6.12-fold expression) and then decreased at D14. Vitoss BA RUNX2 expression trended towards an increase again at both D21 (4.14-fold expression) and D42 (5.80-fold expression). RUNX2 expression in Vitoss BA was higher compared to RUNX2 expression in Vitoss on all observation points, significantly on D42 (p = 0.005).

SPP1 expression in Vitoss scaffolds increased from D7 (0.8-fold expression) to D21 (1.3-fold expression) by 57%, then decreased to D42 (1.0-fold expression) by 21% (Fig 5C). SPP1 expression in Vitoss BA scaffolds decreased from 4.5-fold expression on D7 to 2.4-fold expression on D14 by 47%, then constantly increased to its maximum of 4.6-fold gene expression on D42 (increase of 93%). SPP1 expression levels in Vitoss BA were significantly higher compared to SPP1 expression in Vitoss at all time points (D7: p = 0.005; D14: p = 0.037; D21: p = 0.047; D42: p = 0.028).

Mimicking SPP1 expression over time, COL1A gene expression in Vitoss scaffolds increased from D7 to D21 by 72% and decreased by 63% until D42. COL1A expression in Vitoss BA scaffolds decreased from D7 to D14 by 23%, but then increased significantly (p = 0.013) from D14 to its maximum on D42 by 426% (Fig 5D). On D14, COL1A expression in Vitoss BA was non-significantly lower than in Vitoss, at any other observation point COL1A expression was higher in Vitoss BA, with a significant difference on day 42 (p = 0.005).

## Discussion

The combination of β-TCP and 45S5 BG for application as synthetic bone substitutes might be an attractive way to overcome the limitations by combining the strengths of both material types [6]. However, there is limited evidence concerning the influence of BG particles on the osteogenic properties, on cell vitality and proliferation in β-TCP based bone substitute materials when used as a composite material [6, 31]. Therefore, in this study, the 45S5 BG-supplemented β-TCP based composite bone substitute material Vitoss BA was compared to Vitoss without BG addition in order to assess the influence of 45S5 BG particles on cell vitality, proliferation and osteogenic properties using a variety of *in-vitro* assays. Scaffolds of the same morphology were made from both substitute materials and seeded with MSC before incubation in static cell culture conditions for up to 42 days. Whilst cell count underwent dynamic changes in the Vitoss group, cells seeded on Vitoss BA remained stable during incubation time and cells in both groups exhibited the same level of vitality despite different cell number. Osteogenic differentiation of the MSC was detected in both groups, however, cell differentiation and maturation were faster in Vitoss BA scaffolds as shown by early differences in ALP activity and superior activity of osteogenic target genes, representing an improvement of osteogenic properties through the addition of 45S5 BG particles.

This study was executed with a high grade of standardization as extrinsic culture conditions, scaffold morphology, and cell properties are equal in each group, allowing to focus on the influence of BG particles on the biological properties of the scaffolds in detail. Despite the use of human MSC, transferability of the study remains at the low level of static cell culture conditions: the scaffolds were not exposed to motion, neither fluid movement nor physical load stress.

One limitation in the use of 45S5 BG, at least in *in-vitro* settings, is the high bioreactivity of the glass combined with an initial burst release of Sodium-ions leading to a dramatic increase in local pH [41, 42]. Especially under static culture conditions, 45S5 BG can damage cells, having a negative impact on cell vitality and causing decreased osteogenic differentiation [41]. The pH in the medium surrounding Vitoss BA scaffolds was significantly higher at all time points compared to the medium containing Vitoss scaffolds. The maximum difference was detectable at D7 and then steadily decreased to the pH level of media containing Vitoss scaffolds. It has been demonstrated that the pH-alterations positively correlate with the ion release of the BG: increased ion release causes higher pH [43]. It might therefore be concluded that the maximum ion release of the BG, along with the corresponding strongest biological impact on the MSC population, takes place during the first week of cell culture and progressively decreases after that. However, at all time points, the BG particles continue to release ions, explaining the remaining significant elevation of pH in the media when compared to media containing Vitoss scaffolds. It has been shown that slightly alkaline surroundings favor the function of osteoblasts whilst the resorbing osteoclasts prefer acid environments [44–46]. Osteoclasts are of a certain relevance in the first steps of physiological bone remodeling since they create cavities in bone (or resorbable biomaterials) that serve as docking sites for osteoblasts [47]. Furthermore, osteoclasts release ionized calcium to the extracellular space that is incorporated into the osseous matrix by local osteoblasts [48]. Since the culture type used in this study, consisting of osteoblast precursor cells (MSC in this case), does not include the hematopoietic lineage derived osteoclasts or osteoclast-like cells, only the effect on the “osteoblast part” of bone regeneration can be analyzed [48]. The higher pH in this setting is likely to favor the viability of osteoblasts leading to increased ALP activity and faster maturation of the MSC population towards osteoblasts. However, these effects might be different when using either cell culture approaches that include osteoclast cell lines or *in-vivo* models [48–50]. The ion release from the BG particles in Vitoss BA causes an upregulation of genes correlating with osteogenic differentiation; however, on basis of the data from this project, it cannot be answered if the rising pH, the ion release, or a combination of both is responsible for the changes in cell metabolism.

It has been shown that 3D cultivation of cell-seeded scaffolds under static cell culture conditions is affiliated with a decrease in cell number during the cultivation period [40]. These findings can partially be supported by the data obtained in this study: In both groups, cell number decreases during time, even significantly from D1 to D42. Whilst the maximum decrease was detectable for Vitoss BA scaffolds from D1 to D7, the cell number remained in a steady state from D7 to D42. The cell number in Vitoss scaffolds did not reduce that strongly during the first week compared to Vitoss BA. However, the cell number decreased steadily over time and did not reach a steady state during the incubation period of 42 days (Fig 4B). It seems that after an initial harmful environment is created by the BG particles in Vitoss BA, the trend towards cell death stops. Since the cell number in Vitoss never reached the low level of the cell number in Vitoss BA, it cannot be clarified if the cell number would remain stable in Vitoss scaffolds or would drop even more if the incubation period would have been longer than 42 days. One might therefore conclude that the BG particles allow the sustaining of a stable cell population at a certain level once the initial excessive and (within this *in-vitro* setting) potentially harmful bioactivity of 45S5 BG particles is reduced by contact to cell culture medium [41].

Despite the significantly lower cell population number on D42 compared to D1, as measured using the PicoGreen dsDNA assay, the viability of the cells increased significantly over the same period in both groups. This increase resulted in a significantly higher PrestoBlue reduction at D42 in the Vitoss group compared to Vitoss BA-scaffolds. Resazurin, the blue dye in PrestoBlue reagent, is reduced in the presence of living cells. It is not yet clearly determined whether the reduction is a chemical reaction in the medium or takes part intracellularly with resazurin as an electron acceptor for many enzymes such as the widely distributed diaphorase group [51]. Most likely, both aspects of chemical and enzymatic degradation play a role in PrestoBlue reduction [51]. Since the reduction of the dye increased over the incubation period despite decreasing cell numbers, it can be surmised that both scaffold types exhibit a positive influence on the cell metabolism activity. Assuming that cell number and PrestoBlue reduction are correlated, the cells on Vitoss BA scaffolds seem to exhibit higher metabolic activity during the first weeks of differentiation, whilst with approaching cell number, cells seeded on Vitoss scaffolds show better performance on D42. Another important aspect is the relation of resazurin reduction and grade or level of cellular differentiation: There is some evidence that cell populations such as stem cells and osteoblasts decrease their level of metabolic activity with ongoing differentiation [52–54]. Once osteoblasts become osteocytes, the cells slow down the energy-demanding production of extracellular matrices [53, 54]. qPCR analysis revealed a faster maturation of the MSC in Vitoss BA-scaffolds detected by significantly higher expression patterns of osteogenic genes in the late stages of the incubation period. The higher grade of differentiation might therefore negatively influence cell metabolic activity at D42 in Vitoss BA scaffolds and serves as a possible explanation for the significant differences in PrestoBlue reduction between the two groups.

For visualization purposes, *in-situ* fluorescence microscopy using a live/dead staining was undertaken. Intact cells were clearly visible in both groups (Fig 3E–3H). Some cells in the Vitoss BA group grew around BG particles building cell clusters (Fig 3G and 3H). The BG particles were detectable during the whole incubation period up until day 42. Interestingly, the staining sensitivity of the BG particles decreased over time which can be a result of surface alterations, such as formation of a carbonate-substituted hydroxyapatite-like (HCA) layer on the BG surface–for very similar cell culture settings, it has been proven that HCA formation takes place, however a direct analysis of HCA within this project was not conducted [19, 20]. To prevent any bias caused by morphological differences, mCT analysis of unseeded scaffolds was performed revealing similar scaffold properties regarding scaffold volume.

Both Vitoss and Vitoss BA scaffolds showed increased ALP activity over the incubation time as a correlate of osteogenic differentiation. The MSC in Vitoss BA seem to profit from the initial release of ions coming from the BG particles (which is indirectly confirmed by the pH-alterations) in terms of osteogenic differentiation in the early phase since ALP activity was significantly higher in Vitoss BA compared to Vitoss on D1 and then reached a steady state in both groups from D14 on. The ALP activity measurement is standardized to the cell count [40]. Therefore, the initial cytotoxicity caused by the BG particles, resulting in lower cell numbers, does not have an influence on the outcome of the analysis, since the evaluation method solely reflects the ALP production of the “single cell” [40]. With decreasing BG release over time, ALP activity levels of Vitoss BA remain in a steady state. In combination with the findings obtained from pH analysis, it might be concluded that the initial ion release from the BG particles is favorable for osteogenic differentiation. This correlation was described in literature before [55]. Vitoss BA contains 20 wt% BG particles with a size of 90–150 μm. Since the ratio of volume to surface area is of certain importance for the bioactivity of 45S5 BGs, the osteogenic properties can be tailored by addition of BG particles with different morphology, e.g. towards long-lasting ion release [56, 57].

Quantitative gene expression analysis (qPCR) showed superior osteogenic differentiation of MSC seeded on Vitoss BA compared to Vitoss. RUNX2 is one of the key transcription factors, mainly of importance during the early phase of osteogenic differentiation, since it is upregulated in pre-osteoblasts and downregulated in mature osteoblasts [58]. RUNX2 expression showed a peak on D7 for both groups. Whilst it declined in the Vitoss group continuously over during the incubation period, RUNX2 expression was upregulated again in the Vitoss BA group from D14 on, resulting in significantly higher expression levels on day 42 (Fig 5B). The upregulation of RUNX2 in the late phase might be explained by the ability of osteoblasts to stimulate differentiation of undifferentiated cells in their surrounding leading to autocrine and paracrine stimulation of osteoblast activity and differentiation [47, 59, 60]. The stimulation of undifferentiated MSC towards osteoblasts will result in higher RUNX2 expression levels at a later stage amongst the cell population. Furthermore, RUNX2 plays an important role not only in the early but also in the late phase of osteogenesis. Firstly, in the early osteoblast and MSC, RUNX2 expression is upregulated mostly by external stimulation [47, 58]. Since no other external stimulus was added in this study, the early external stimulation most likely comes from the 45S5 BG particles, explaining the superior RUNX2 expression on day 7 in the Vitoss BA group [14]. At a later stage, during the maturation phase of the osteoblasts, RUNX2 is relevant to enhance growth factor expression leading to both further maturation of the respective cell and stimulation of undifferentiated cells towards osteogenic differentiation, which is closing the loop of autocrine and paracrine stimulation [47, 61]. Therefore, the results obtained in this study are most likely to be representative for (1) upregulation of RUNX2 during the differentiation of MSC from the first days in culture induced by the 45S5 BG particles and (2) for the MSC being stimulated towards osteogenic differentiation by autocrine and paracrine mechanisms later during the incubation period compensating the reduced bioactivity of the 45S5 BG particles. One of the most important parts of the organic extraosseous matrix is collagen type I, which is encoded by the COL1A gene [62]. Expression of the COL1A gene is mostly occurring during the shift from early to mature stages of osteoblast maturation when the osteoblasts start building the extracellular matrix [62, 63]. COL1A expression underwent upregulation in Vitoss BA scaffolds during the incubation time with significantly higher expression on D42 compared to the Vitoss group. It has been demonstrated that 45S5 BG enhances the gene expression of COL1A and therefore supports the formation of an extracellular osseous matrix which can be confirmed by the findings in this study [14]. The SPP1 gene encodes for osteopontin, a protein that is relevant for the mineralization of the extracellular osseous matrix [64, 65]. SPP1 gene expression is representative for a later stage of osteoblast differentiation and was found to be significantly higher in Vitoss BA scaffolds for all time points with a maximum on D42. Since SPP1 gene activity is correlated with the maturation of osteoblasts, higher expression patterns are indicative for further developed cell populations. 45S5 BG builds a HCA layer in physiological liquids, which contains calcium, providing not only bonding to surrounding tissues or cells, but also enhanced calcification of the extracellular portion of bone [19]. It was shown that SPP1 gene expression patterns are upregulated by elevated extracellular calcium levels serving as a possible explanation for the simulation of SPP1 gene expression within the Vitoss BA group [66].

Gene expression analysis revealed superior osteogenic properties of Vitoss BA linked to a faster and more effective differentiation and maturation of MSC towards osteoblasts, as confirmed by the elevated activity of COL1A and SPP1. Once the activity of the BG particles decreased, the local cell population sustained a steady state of osteogenic activity, including the stimulation of less or undifferentiated cells towards bone formation. These findings are supported by the RUNX2 gene expression, as well as the ALP activity assessment.

## Conclusions

In this study, pure β-TCP-based Vitoss was compared to its 45S5 BG supplemented composite variant, Vitoss BA, in order to focus on the influence of the BG particles on cell vitality, proliferation, and osteogenic differentiation. The obtained results suggest that 45S5 BG particles exhibit (limited) cell toxic conditions during the first week of incubation, but positively influenced the osteogenic differentiation of MSC and, taking the lower cell count into account, did not have a negative impact on cell vitality. Most of the stimulatory effects induced by the BG particles in Vitoss BA took place during the first week of incubation. In order to achieve a more sustainable stimulation, particles exhibiting another surface-area-to-volume ratio might be added and should undergo consideration in further experimental evaluation. The methodological approach used in this study was designed in order to focus specifically on the influence of the BG particles, using statically cultured osteoblast precursor cells to evaluate these properties by a variety of *in-vitro* methods. However, the findings of this study should be validated using other experimental approaches, for example *in-vivo* protocols that cover and evaluate further relevant characteristics for bone substitutes such as the influence of additional cell types (e.g. osteoclasts) or the impact of the BG particles on vascularization in a more physiological surrounding.
